# Human Sperm Interaction with *Staphylococcus aureus*: A Molecular Approach

**DOI:** 10.1155/2012/816536

**Published:** 2012-10-15

**Authors:** Sonia Gupta, Vijay Prabha

**Affiliations:** Department of Microbiology, Panjab University, Chandigarh 160014, India

## Abstract

Sperm immobilization factor (SIF) causing 100% immobilization of spermatozoa isolated from *Staphylococcus aureus* when characterized using LC-MS (Liquid chromatography-mass spectrometry) showed that this 20 kDa protein had peptide sequence similarity with hsp-70 protein. It was found to completely (100%) inhibit Mg^++^ ATPase activity of spermatozoa at concentration of 100 **μ**g mL^−1^. Sperm samples treated with SIF also showed reduction in calcium ionophore-induced acrosome reaction as compared to control samples (treated with calcium ionophore alone). Binding studies of FITC labelled SIF with spermatozoa using fluorescent microscopy showed binding of SIF to the surface of spermatozoa indicating the presence of SIF binding receptor. The receptor was extracted by 3M NaCl and purified by gel permeation chromatography. Characterization of the receptor by MALDI-TOF (Matrix-assisted laser desorption ionization-time of flight) indicated that the receptor shared sequence similarity with MHC class II antigen. A calorimetric study showed that the receptor moiety on spermatozoa was specific for the purified ligand as binding of the receptor to ligand was enthalpically (−11.9 kJ mole^−1^) as well as entropically (21.53 J mole^−1^ K^−1^) favored resulting in the Gibb's free energy of −18.57 kJ mole^−1^.

## 1. Introduction

*Staphylococcus aureus* is amongst the most versatile and successful of the human pathogens. It has the ability to cause a variety of infections in numerous ecological niches within the host. It colonizes the nares, axillae, vagina, pharynx, or damaged skin surfaces and causes a variety of suppurative (pus-forming) infections and toxinoses in humans. Besides this, *S. aureus *is arguably the dominant organism implicated in primary infertility, among males and females alike [1]. *S. aureus *has been observed as causative organism accounting for 68.2% of seminal fluid infections [2]. This is consistent with that reported by Okon et al., where *S. aureus* was isolated from 62.5% of the seminal fluids [3].* S. aureus* has also been reported to be commonly isolated microorganism from cervical samples [4]. Huwe et al. studied the influence of different uropathogenic microorganisms on human sperm motility parameters by means of CASA and reported that *S. aureus* retards the sperm motility [5]. Similar studies were done by Liu et al. on the effect of certain uropathogenic microorganisms on human sperm motility parameters and found significant decrease in sperm motility when spermatozoa were coincubated with *S. aureus *[6]. Some authors have suggested that direct interaction between bacteria and spermatozoa facilitates immobilization of spermatozoa [7–9], while others have reported evidence for soluble spermicidal factor produced and secreted by bacteria in the extracellular medium [10]. 

In an earlier work done in our laboratory, we have also been able to isolate a strain of *S. aureus* from cervix of an infertile woman, causing 100% immobilization of spermatozoa without agglutination. The factor responsible for sperm immobilization was isolated and purified [11]. Further, its effect on various sperm parameters was studied. Motility of spermatozoa requires energy in the form of ATP [12] that is provided by sperm mitochondria to power the flagellar motion that propels the sperm to the site of fertilization [13]. It thus seems possible that the inhibition of sperm motility after treatment with SIF may be due to decrease in the ATPase activity or imbalance in the molecules of biological significance. Further, the analysis of acrosome reaction in human sperm is recommended to estimate the fertilization ability of human sperm in the clinical field [14]. Hence, the effect of SIF on Mg^++^ATPase activity and acrosome reaction was evaluated. Then an attempt was made to isolate the corresponding receptor for SIF [15]. Further characterization of both SIF and SIF binding receptor will help in better understanding of SIF and receptor interactions which can be aimed as a potential candidate for treatment of unexplained infertility.

## 2. Materials and Methods

### 2.1. Semen Samples

Semen samples were obtained from males attending infertility clinic at Government Multispeciality Hospital, Sector 16, Chandigarh, by masturbation into sterile wide mouth container. Only ejaculates showing normal semen parameters according to WHO criteria [16] were used. Samples underwent liquefaction at room temperature for 30 mins. Experiments were performed within 1 h of obtaining samples. A preparation of washed sperm samples was done, in which the sperm cell pellet was retained after centrifugation at 500 rpm for 10 min and thereafter it was washed twice with sterile phosphate buffer saline (PBS) (50 mM, pH 7.2).

### 2.2. Microorganism

Cervical swabs were taken from 16 women below the age of 35 years, suffering from unexplained infertility attending the Department of Obstetrics and Gynaecology at Government Multispeciality Hospital, Sector 16, Chandigarh. Samples were streaked on blood agar plates and the plates were incubated aerobically at 37°C for 24–48 h. Isolates were identified according to the Bergey's Manual of Determinative Bacteriology. Microorganisms were identified up to genus level only. In total, 27 isolates were obtained. Amongst the various isolates, *Staphylococcus *(51.85%) was the predominant organism present. When the effect of 24 and 48 h old cell culture and cell free supernatant of cervical isolates on human sperm motility was studied *in vitro*, the results showed that 17 out of 27 isolates (63%) significantly decreased sperm motility [17]. One of the isolate causing 100% immobilization of spermatozoa was selected for further studies. Only this strain was further identified by biochemical tests and was found to be coagulase positive *Staphylococcus aureus. *This strain has been designated as *S. aureus* VP2. 

### 2.3. Isolation and Purification of SIF from Bacteria

SIF was extracted and purified from 72 h old cell culture of* S. aureus *by the method previously standardized in the laboratory [11]. Briefly, the cell culture of *S. aureus *grown in brain heart infusion (BHI) broth, under shake conditions (220 rpm) at 37°C for 72 h, was centrifuged at 5,000 rpm for 15 min at 4°C. SIF was purified from the supernatant by ammonium sulphate precipitation, gel permeation chromatography, and ion exchange chromatography. To check the purification status, polyacrylamide gel electrophoresis (PAGE) was carried out.

### 2.4. Liquid Chromatography-Mass Spectrometry (LC-MS) of Purified SIF

#### 2.4.1. Processing of Bands and Tryptic Digestion

Following PAGE, protein band was excised and gel piece was washed once with 100 *μ*L HPLC grade water for 10 min and then destained in 100 *μ*L of destaining solution 1 (50% 100 mM ammonium bicarbonate in 50% acetonitrile) by incubating at 37°C for 30 min. Destaining solution was discarded and gel piece was dehydrated in 100 *μ*L of acetonitrile (ACN) at 37°C for 5 min and then air dried for 5 min at 37°C. The gel piece was reduced in 150 *μ*L of 10 mM of Dithiothreitol (DTT)/100 mM of ammonium bicarbonate by incubating at 56°C for 30 min. It was then alkylated in 100 *μ*L of 50 mM iodoacetamide/100 mM ammonium bicarbonate in dark for 30 min and alkylation buffer was discarded. Gel piece was washed with 100 *μ*L destaining solution, removed, and again dehydrated with 100 *μ*L ACN for 5 min and then air dried at 37°C for 5 min. Gel piece was rehydrated in ~5 *μ*L trypsin solution (20 ng *μ*L^−1^) for 10 min at 37°C. Gel piece was incubated at 37°C for 16 h. 

#### 2.4.2. Peptide Extraction

To extract the peptides after the overnight incubation, ~20 *μ*L peptide extraction solution (1% Trifluoroacetic acid, TFA) was added and sonicated in a water bath sonicator for 5 min. 1–5 *μ*L of the extracted peptides were used for subsequent MS analysis.

#### 2.4.3. LC-MS

A 6 *μ*L solution was injected for analysis with LC-MS/MS (Agilent, Palo Alto, CA, USA). Data was analyzed using Agilent Ion Trap Analysis software version 5.2 and proteins were identified by database search against the MASCOT database.

### 2.5. *In Vitro* Effect of SIF on Sperm Mg^++^ATPase Activity

Mg^++^ ATPase activity of spermatozoa was studied according to Kielley [18] and Chappel [19] with slight modifications. Tris-HCl (0.2M, pH 7.6) washed spermatozoa (1 × 10^8^/mL) were sonicated at 50 Hz for 10 min (10 cycles of 30s with 1 min interval) at 4°C. The reaction mixture for ATPase consisted of 0.2 mL Tris-HClbuffer (0.2M, pH 7.6), 0.2 mL of MgCl_2_ (5 mM), 0.2 mL of ATP (6 mg mL^−1^), and 0.2 mL of sonicated sperm suspension. Different concentrations of SIF (12.5 *μ*g, 25 *μ*g, 50 *μ*g, and 100 *μ*g) were added to the reaction mixture. The mixture was incubated at 37°C for 1 h. After this period of incubation, the reaction was stopped by adding 1 mL of cold 10% Trichloroacetic acid (TCA) and then incubated at 4°C overnight for protein precipitation. The control tubes contained all the components of the reaction mixture but TCA was added in the beginning to stop the ATPase activity. Inorganic phosphorus (Pi) released was determined according to the method of Boyce et al. [20]. One unit of ATPase is expressed as *μ*g of the Pi released after 1 h of incubation. The experiment was repeated thrice.

### 2.6. *In Vitro* Effect of SIF on Acrosome Reaction of Spermatozoa

The semen sample after liquefaction was washed twice (500 rpm, 5 min) with human tubal fluid medium (HTFM) containing 1% human serum albumin (HSA) [21]. Sperm concentration was adjusted to 40 × 10^6^ mL^−1^ and sperm suspension was divided into two aliquots (test and control). The test sample was incubated for 3 h at 37°C with equal volumes of SIF, whereas the control was prepared by adding equal volume of PBS (50 mM, pH 7.2) under same conditions. After 3 h, spermatozoa from both test and control were treated with either 0.1% DMSO (spontaneous acrosome reaction) or 10 *μ*M calcium ionophore A23187 (induced acrosome reaction) for 1 h at 37°C. Subsequently, spermatozoa were washed in HTFM without HSA (500 rpm, 10 min). The pellet was resuspended in 3% glutaraldehyde and incubated for 20 min at 37°C. After further washing the pellet was resuspended in 10–50 *μ*L HTFM and smeared onto a slide. After drying, spermatozoa were stained in Bismark brown (0.8% in deionized water, pH 1.8) for 5 min, washed three times with water, and stained with Rose Bengal (0.8% in 0.1M Tris buffer, pH 5.6) for 25 min. After second washing procedure, spermatozoa were dehydrated in 50%, 70%, and 96% ethanol rinsed with water, and examined at 1000x magnification under light microscope.

### 2.7. Binding Studies of SIF to Spermatozoa by Fluorescent Microscopy

Purified ligand was conjugated to FITC using FITC labeling kit (GeNei FITC Labelling Kit Bangalore Genei (India) Pvt. Ltd.). 1 mg of purified ligand was mixed with FITC according to F/P ratio as per the instructions given in the kit.

Liquefied semen sample was washed twice with PBS and the pellet was finally suspended in 500 *μ*L of PBS. To 100 *μ*L of sperm suspension, 200 *μ*L of conjugated ligand was added and incubated at 37°C for 1 h. Then 150 *μ*L of 3% formaldehyde was added and the reaction mixture was incubated at 37°C for 1 h. After incubation, the reaction mixture was washed thrice with PBS. The pellet was finally dissolved in 50 *μ*L of PBS (50 mM, pH 7.2). A wet mount was prepared and observed under fluorescent microscope 1000x magnification.

### 2.8. Extraction of Receptor for SIF from Human Sperm

SIF receptor was extracted from human spermatozoa by the method previously standardized in the laboratory [15]. Briefly, the receptor was extracted by treating washed spermatozoa with 3M NaCl for 4 h at 37°C under shaking conditions. 3M NaCl-treated sperm sample was centrifuged at 1500 rpm for 15 min and then dialyzed extensively against PBS (50 mM, pH 7.2) under cold conditions. It was concentrated using polyethylene glycol (PEG 6000). The purification of the receptor was further done using gel filtration chromatography and the purity of this preparation was checked by native PAGE. 

### 2.9. Matrix-Assisted Laser Desorption/Ionization Time of Flight (MALDI-TOF) of Receptor

Processing of protein bands, tryptic digestion, and peptide extraction was done in the same way as described earlier for LC-MS of SIF. sample for MALDI-TOF analysis was prepared using dried droplet method. 1 *μ*L peptide solution (peptide extracts after tryptic digestion) and 1 *μ*L of a suitable matrix, for example, alpha-cyano hydroxycinnamic acid (HCCA) in 1 : 2 v/v of acetonitrile (ACN): 0.1% TFA, were mixed nicely. 1 *μ*L of this mixture was spotted on a MALDI target plate and allowed to air dry at room temperature. Peptide calibration standard (BRUKER) was also prepared in the same way. MALDI target plate was loaded into Ultraflex MALDI-TOF for subsequent peptide spectra acquisition and analysis. A LASER power of 337 nm wavelength was used for ionization of the samples spotted on the target plate. Peptide peaks were calibrated with peaks obtained from the peptide calibration standard. After peptide spectra were obtained, MS analysis was carried out using Flex analysis software (v 2.2, BRUKER). Subsequent MS data analysis was carried out using Biotools software (v 2.2, BRUKER) and MASCOT search engine (Matrix Science) against the NCBI database.

### 2.10. Calorimetric Analysis of Receptor-Ligand Interaction

The enthalpy of binding of ligand with the receptor was determined in PBS using a microreaction calorimeter. An amount of 1.5 mL of receptor (200 *μ*g mL^−1^) in buffer was placed in each of the calorimetric vials and 250 *μ*L syringe (speed 3 *μ*L sec^−1^) was loaded with the ligand (500 *μ*g mL^−1^). The experiment was conducted using the titration mode. The binding constant, K, and enthalpy of binding, Δ*H*° were computed by using iterative nonlinear least square regression method.

## 3. Results

*S. aureus *isolated from the cervix of a woman with unexplained infertility inhibited sperm motility by secreting the factor extracellularly. Further, the factor was isolated and purified as previously reported. Purified SIF was a protein of about 20 kDa with remarkable sperm immobilization activity.

### 3.1. LC-MS of Purified SIF

According to LC-MS measurements of the 20 kDa band, the protein showed the peptide sequence similarity with hsp-70 protein when matched with proteins in the NCBInr database, using the Mascot search program. The search yielded a top score of 143 for hsp-70 like protein as shown by the histogram of the score distribution for the 20 best matching proteins. Protein scores greater than 57 (*P* < 0.05) were considered to be significant.

### 3.2. *In Vitro* Effect of SIF on Sperm Mg^++^ ATPase Activity

The effect of purified SIF from *S. aureus* on the Mg^++^ adenosine triphosphatase activity of pooled and sonicated spermatozoa from human was studied. From the results (Table 1), it could be observed that the SIF inhibited Mg^++^ ATPase activity of spermatozoa in dose-dependent manner. At a concentration of 100 *μ*g mL^−1^ of SIF, there was no detectable Mg^++^ ATPase activity, whereas at lower concentration of 50 *μ*g mL^−1^ of SIF, ATPase activity decreased from 931.7 ± 1.3 units (control) to 195.23 ± 2.3 units. 

### 3.3. *In Vitro* Effect of SIF on Sperm Acrosome Reaction

**Figure 1:**
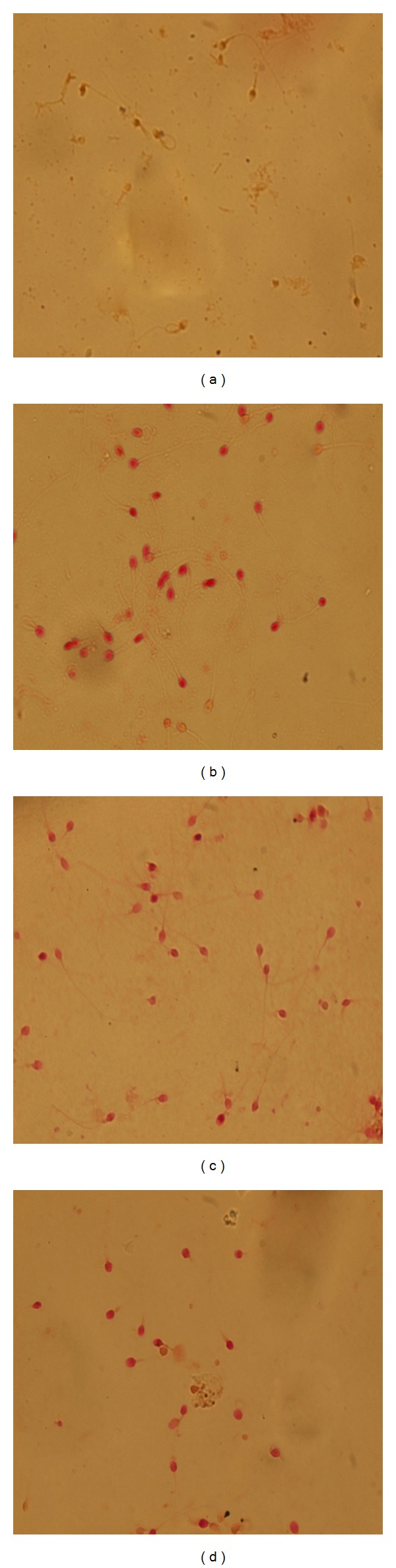
Red or pink staining of the acrosomal region indicated intact acrosomes, whereas white, brown, or yellowish acrosomes were interpreted as acrosome reacted. Induced sperm acrosome reaction on treatment with calcium ionophore (1000x)-positive control (a). Effect of SIF on calcium ionophore induced sperm acrosome reaction (1000x) (b). Sperm acrosome reaction on treatment with DMSO (1000x)-negative control (c). Effect of SIF on sperm acrosome reaction in DMSO-treated sample (1000x) (d).

When the effect of SIF on human sperm acrosome reaction was studied two patterns of staining were observed under light microscope (1000x). Red or pink staining of the acrosomal region indicated intact acrosomes, whereas white, brown, or yellowish acrosomes were interpreted as acrosome-reacted. The percentages of spontaneously acrosome reacted spermatozoa in SIF-treated samples were comparable to control (DMSO). On treatment with calcium ionophore, majority (approx. 100%) of spermatozoa underwent acrosome reaction. However, spermatozoa incubated with SIF in HTFM (with 1% HSA) failed to undergo acrosome reaction in response to calcium ionophore challenge (Figure 1).

### 3.4. Binding Studies of SIF with Spermatozoa by Fluorescent Microscopy

**Figure 2:**
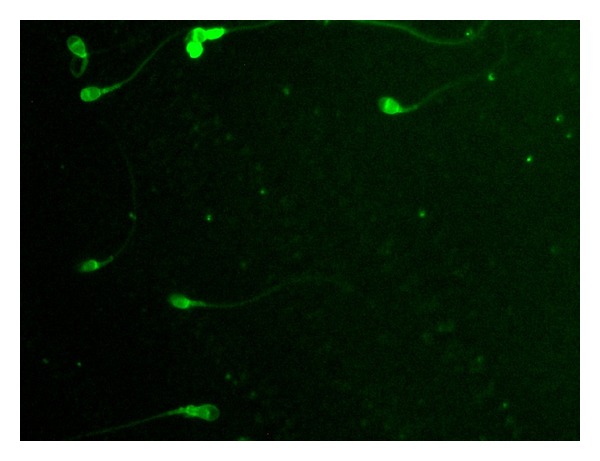
Fluorescent microscopy of human spermatozoa incubated with FITC labelled SIF 1000x.

When sperm samples treated with FITC labelled SIF were observed under fluorescent microscope, bright green fluorescence was seen over the spermatozoa which depicts the binding of SIF with spermatozoa. This indicates that some receptors might be present on the surface of spermatozoa to which SIF binds (Figure 2). 

### 3.5. MALDI-TOF of Purified Receptor

According to MALDI-TOF analysis of the purified 62 kDa band, the protein showed the peptide sequence similarity with MHC class II antigen when the resulting spectrum was used to search for matching proteins in the NCBInr database, using the Mascot search program. The search yielded a top score of 68 for MHC class II antigen (protein scores greater than 66 are significant; *P* < 0.05). 

### 3.6. Calorimetric Study of Receptor-Ligand Interaction

The binding constant, K (1350/M), and enthalpy of binding, Δ*H*°, (−11.9 kJ mole^−1^) were computed from the experimentally calculated enthalpy of interaction between ligand and receptor, using iterative nonlinear least square regression method (Table 2). The values of free energy and entropy were found to be −18.57 kJ mole^−1^ and 21.53 J mole^−1^K^−1^, respectively, calculated from the following equations:

(1) $$\begin{array}{c} \Delta G^{{}^{\circ}}=-\;RT\ln K,\\ \Delta S^{{}^{\circ}}=\frac{\left(\Delta H^{{}^{\circ}}-\Delta G^{{}^{\circ}}\right)}{T}. \end{array}$$

## 4. Discussion

*S. aureus *is an aggressive, opportunistic Gram-positive pathogen that colonizes the skin, anterior nares, axillae, pharynx, and urogenital tract of 20% of healthy humans. Jiang and Lu reported *S. aureus *as the predominant flora in infertile men with a significant decrease in sperm motility [22]. 

In an earlier work done in our laboratory, *S. aureus* causing immobilization of human spermatozoa was isolated from the cervix of a woman with unexplained infertility, and a sperm immobilization factor (SIF) was purified. Further characterization was done using LC-MS. LC-MS studies indicated that SIF showed peptide sequence similarity with hsp-70 protein. Neuer et al. [23] reported that heat shock proteins (HSP) are immunodominant antigens of numerous microbial pathogens, for example, *Chlamydia trachomatis*, which have been recognized as the main cause of infertility [23]. 

To further elucidate the contribution of SIF to reproductive failure, the effect of purified SIF from *S. aureus* on the Mg^++^ ATPase activity of spermatozoa was studied and the results showed that SIF decreased the ATPase activity. There was no detectable Mg^++^ ATPase activity when spermatozoa were incubated with SIF at concentration of 100 *μ*g mL^−1^. Any agent affecting ATPase activity of sperms affects the motility of spermatozoa making it incapable of fertilization. For example, in case of sea urchin spermatozoa, it was observed that motile sperm had an ATPase activity of 0.16 *μ*M Pi (min × mg protein)^−1^, while sperm that had been rendered nonmotile by homogenizing had an activity of 0.045 *μ*M Pi (min × mg protein)^−1^ [24], suggesting inhibition of Mg^++^-dependent ATPase could be one of the possible mechanisms of sperm immobilization.

Further, the evaluation of acrosome reaction can be used to predict fertilization success. Although the rate of spontaneous acrosomal reaction of spermatozoa does not correlate with the success rate of *in vitro* fertilization (IVF), the index of inducibility, measured by the difference between induced (after incubation with calcium ionophore or exposure to low temperature) and spontaneous acrosomal reaction, is of prognostic value for sperm fertilization capacity [25]. When the effect of SIF on human spermatozoa was studied, it was found that the percentages of acrosome-reacted spermatozoa obtained in tests treated with SIF were consistently lower than those obtained in calcium ionophore-treated samples (positive control). Bacteria including *Ureaplasma urealyticum *and *Mycoplasma hominis* have been demonstrated to have a negative effect on ionophore-induced acrosomal reaction of human spermatozoa *in vitro* [26, 27]. El-Mulla et al. also studied the effect of *E. coli* on acrosomal reaction of spermatozoa [28]. It was shown that the inducibility of the acrosome reaction was significantly lower in semen samples pretreated with *E. coli* than in the control samples. The results demonstrated that *E. coli* affect the inducibility of the acrosome reaction *in vitro* and may impair the fertilizing capacity of human spermatozoa. Several studies have found that sperm responsiveness to acrosome reaction inducers is reduced in infertile patients [29]. Therefore, SIF inhibiting the induction of acrosome reaction may be impairing the fertilizing capacity of human spermatozoa.

From the results it appears that the effect of SIF on inhibition of Mg^++^ ATPase may be separate from ionophore mediated acrosome reaction as disrupted spermatozoa were used for the estimation of Mg^++^ ATPase activity while intact spermatozoa were evaluated for acrosome reaction.

Further, when the fluorescent microscopy of spermatozoa incubated with FITC labelled SIF was done, bright green fluorescence was seen over the spermatozoa surface. This binding between SIF and spermatozoa gives direct evidence that some kind of receptors might be present on the surface of spermatozoa to which labelled SIF binds. Therefore, in the present study, an attempt was made to extract, purify, and characterize the receptor from spermatozoa that is responsible for its interaction with SIF produced by *S. aureus*. MALDI-TOF studies of receptor indicated that receptor shows sequence similarity with MHC class II antigen when matched with NCBInr database. These results are in agreement with those of earlier studies by Morimoto et al. who clearly demonstrated the expression of the major histocompatibility complex (MHC) class II molecules on murine sperm cells by means of radioimmunoassay as well as by enzyme immunoassay [30]. The study revealed that the site of sperm for binding foreign DNA was mediated by the complex structure of the MHC class II molecules localized at the posterior region of sperm head.

Ligand association with the receptor typically involves changes in the intramolecular and intermolecular interactions and dynamics of the system components. The changes in bonding interactions that occur upon ligand binding are reflected in the reaction enthalpy and entropy, which in turn determine the free energy of receptor-ligand association. Microcalorimetric instruments and methods are essential tools for the general understanding of binding thermodynamics of biological macromolecules and specific biological systems. In the present study, when microreaction calorimeter was used to determine the free energy (Δ*G*), enthalpy (Δ*H*), and entropy (Δ*S*) of binding of SIF with purified receptor it was observed that reaction was exothermic as heat was produced which indicated specific interaction between receptor and purified SIF.

From the above preliminary observations, it can be concluded that SIF isolated from *S. aureus* could bind to spermatozoa and sperm surface protein might act as receptor for binding to SIF. The study also indicates that receptor-ligand interaction might be responsible for immobilization of spermatozoa by SIF. Thus, the future studies of detailed molecular mechanisms of sperm immobilization by these receptor-ligand interactions could possibly lead to useful insights for improvements in the treatment of infertility, and in attempts to create safe as well as effective sperm immobilizing contraceptives.

## Figures and Tables

**Table 1:** Effect of SIF on Mg^++^ ATPase activity.

*S*. number	Concentration of SIF (*μ*g mL^−1^)	Mg^++^ ATPase activity (units)*
Control	—	931.7 ± 1.3
1	12.5	712.86 ± 2.3
2	25.0	574.76 ± 0.96
3	50.0	195.23 ± 2.3
4	100.0	0.004 ± 0.0025

*Each value is the mean ± S.D of three observations.

**Table 2:** Heat released on receptor ligand interaction.

*S*. number	Conc. of ligand (*μ*M)	Conc. of receptor (*μ*M)	Heat released (mJ)
1	7.5 × 10^−4^	4.8 × 10^−3^	9.7
2	15.0 × 10^−4^	4.8 × 10^−3^	9.0
3	22.5 × 10^−4^	4.8 × 10^−3^	8.6
4	30.0 × 10^−4^	4.8 × 10^−3^	5.6
5	37.5 × 10^−4^	4.8 × 10^−3^	2.3
6	45.0 × 10^−4^	4.8 × 10^−3^	2.1
7	52.5 × 10^−4^	4.8 × 10^−3^	2.0

## References

[1] Momoh ARM, Idonije BO, Nwoke EO (2011). Pathogenic bacteria-a probable cause of primary infertility among couples in Ekpoma. *Journal of Microbiology and Biotechnology Research*.

[2] Emokpae MA, Uadia PO, Sadiq NM (2009). Contribution of bacterial infection to male infertility in Nigerians. *Online Journal of Health and Allied Sciences*.

[3] Okon KO, Nwaogwu M, Zailani SO, Chana C (2005). Pattern of seminal fluid indices among infertile male partners attending the infertility clinic of University of Maiduguri Teaching Hospital, Maiduguri, Nigeria. *Highland Medical Research Journal*.

[4] Okonofua FE, Ako-Nai KA, Dighitoghi MD (1995). Lower genital tract infections in infertile Nigerian women compared with controls. *Genitourinary Medicine*.

[5] Huwe P, Diemer T, Ludwig M, Liu J, Schiefer HG, Weidner W (1998). Influence of different uropathogenic microorganisms on human sperm motility parameters in an in vitro experiment. *Andrologia*.

[6] Liu JH, Li HY, Cao ZG, Duan YF, Li Y, Ye ZQ (2002). Influence of several uropathogenic microorganisms on human sperm motility parameters in vitro. *Asian Journal of Andrology*.

[7] Diemer T, Weidner W, Michelmann HW, Schiefer HG, Rovan E, Mayer F (1996). Influence of Escherichia coli on motility parameters of human spermatozoa in vitro. *International Journal of Andrology*.

[8] Núñez-Calonge R, Caballero P, Redondo C, Baquero F, Martínez-Ferrer M, Meseguer MA (1998). Ureaplasma urealyticum reduces motility and induces membrane alterations in human spermatozoa. *Human Reproduction*.

[9] Erbengi T (1993). Ultrastructural observations on the entry of Chlamydia trachomatis into human spermatozoa. *Human Reproduction*.

[10] Paulson JD (1977). Isolation of a spermatozoal immobilization factor from Escherichia coli filtrates. *Fertility and Sterility*.

[11] Prabha V, Gupta T, Kaur S, Kaur N, Kala S, Singh A (2009). Isolation of a spermatozoal immobilization factor from Staphylococcus aureus filtrates. *Canadian Journal of Microbiology*.

[12] Nelson MF (1980). Wrongful life: impaired infant’s cause of action recognized—Curlender v. Bio-Science Laboratories. *Brigham Young University law review*.

[13] Alcivar AA, Hake LE, Millette CF, Trasler JM, Hecht NB (1989). Mitochondrial gene expression in male germ cells of the mouse. *Developmental Biology*.

[14] Ohashi K, Saji F, Kato M, Tsutsui T, Tomiyama T, Tanizawa O (1995). Acrobeads test: a new diagnostic test for assessment of the fertilizing capacity of human spermatozoa. *Fertility and Sterility*.

[15] Prabha V, Chaudhary N, Kaur S (2011). Molecular mimicry between bacteria and spermatozoa. *Journal of Urology*.

[16] World Health Organisation (2010). *WHO Laboratory Manual For the Examination and Processing of Human Semen*.

[17] Prabha V, Aanam, Dhir T, Kaur S (2011). Bacteriological study of the cervix of females suffering from unexplained infertility. *American Journal of Biomedical Sciences*.

[18] Kielley MW, Colowick SP, Kaplan NO (1955). Mitochondrial ATPase. *Methods in Enzymology*.

[19] Chappel JB (1963). The effect of alkylguanidines on mitochondrial metabolism. *The Journal of Biological Chemistry*.

[20] Boyce A, Casey A, Walsh G (2004). A phytase enzyme-based biochemistry practical particularly suited to students undertaking courses in biotechnology and environmental science. *Biochemistry and Molecular Biology Education*.

[21] Quinn P, Kerin JF, Warnes GM (1985). Improved pregnancy rate in human in vitro fertilization with the use of a medium based on the composition of human tubal fluid. *Fertility and Sterility*.

[22] Jiang J, Lu DY (1996). Detection of bacteria from semen of infertile males and their seminal parameters. *Chinese Journal of Andrology*.

[23] Neuer A, Spandorfer SD, Giraldo P, Dieterle S, Rosenwaks Z, Witkin SS (2000). The role of heat shock proteins in reproduction. *Human Reproduction Update*.

[24] Gibbons BH, Gibbons IR (1972). Flagellar movement and adenosine triphosphatase activity in sea urchin sperm extracted with triton X-100. *Journal of Cell Biology*.

[25] Henkel R, Muller C, Miska W, Gips H, Schill WB (1993). Determination of the acrosome reaction in human spermatozoa is predictive of fertilization in vitro. *Human Reproduction*.

[26] Rose BI, Scott B (1994). Sperm motility, morphology, hyperactivation, and ionophore-induced acrosome reactions after overnight incubation with mycoplasmas. *Fertility and Sterility*.

[27] Kohn FM, Erdmann I, Oeda T, El-Mulla KF, Schiefer HG, Schill WB (1998). Influence of urogenital infections on sperm functions. *Andrologia*.

[28] El-Mulla KF, Köhn FM, Dandal M (1996). In vitro effect of Escherichia coli on human sperm acrosome reaction. *Archives of Andrology*.

[29] Krausz C, Bonaccorsi L, Luconi M (1995). Intracellular calcium increase and acrosome reaction in response to progesterone in human spermatozoa are correlated with in-vitro fertilization. *Human Reproduction*.

[30] Morimoto RI, Tissieres A, Georgopoulos C, Morimoto RI, Tissieres A, Georgopoulos C (1990). The stress response, function of the proteins, and perspectives. *Stress Proteins in Biology and Medicine*.

